# Atherosclerosis Linked to Aberrant Amino Acid Metabolism and Immunosuppressive Amino Acid Catabolizing Enzymes

**DOI:** 10.3389/fimmu.2020.551758

**Published:** 2020-09-28

**Authors:** Bozidarka L. Zaric, Jelena N. Radovanovic, Zoran Gluvic, Alan J. Stewart, Magbubah Essack, Olaa Motwalli, Takashi Gojobori, Esma R. Isenovic

**Affiliations:** ^1^Department of Radiobiology and Molecular Genetics, “VINČA” Institute of Nuclear Sciences - National Institute of the Republic of Serbia, University of Belgrade, Belgrade, Serbia; ^2^Department of Endocrinology and Diabetes, Faculty of Medicine, University Clinical-Hospital Centre Zemun-Belgrade, University of Belgrade, Belgrade, Serbia; ^3^School of Medicine, University of St Andrews, St Andrews, United Kingdom; ^4^Computer, Electrical and Mathematical Sciences and Engineering Division (CEMSE), Computational Bioscience Research Center, Computer (CBRC), King Abdullah University of Science and Technology (KAUST), Thuwal, Saudi Arabia; ^5^College of Computing and Informatics, Saudi Electronic University (SEU), Medina, Saudi Arabia; ^6^Biological and Environmental Sciences and Engineering Division (BESE), King Abdullah University of Science and Technology (KAUST), Thuwal, Saudi Arabia

**Keywords:** amino acid, tryptophan, arginine, branched-chain amino acids, metabolism, atherosclerosis, amino acids

## Abstract

Cardiovascular disease is the leading global health concern and responsible for more deaths worldwide than any other type of disorder. Atherosclerosis is a chronic inflammatory disease in the arterial wall, which underpins several types of cardiovascular disease. It has emerged that a strong relationship exists between alterations in amino acid (AA) metabolism and the development of atherosclerosis. Recent studies have reported positive correlations between levels of branched-chain amino acids (BCAAs) such as leucine, valine, and isoleucine in plasma and the occurrence of metabolic disturbances. Elevated serum levels of BCAAs indicate a high cardiometabolic risk. Thus, BCAAs may also impact atherosclerosis prevention and offer a novel therapeutic strategy for specific individuals at risk of coronary events. The metabolism of AAs, such as L-arginine, homoarginine, and L-tryptophan, is recognized as a critical regulator of vascular homeostasis. Dietary intake of homoarginine, taurine, and glycine can improve atherosclerosis by endothelium remodeling. Available data also suggest that the regulation of AA metabolism by indoleamine 2,3-dioxygenase (IDO) and arginases 1 and 2 are mediated through various immunological signals and that immunosuppressive AA metabolizing enzymes are promising therapeutic targets against atherosclerosis. Further clinical studies and basic studies that make use of animal models are required. Here we review recent data examining links between AA metabolism and the development of atherosclerosis.

## Introduction

With the identification of numerous new therapeutic agents and improved medical technology, the last decade has seen a notable advancement toward the prevention and treatment of atherosclerosis. However, despite such signs of progress associated with the treatment and management of atherosclerosis and cardiovascular diseases (CVDs), the mortality and prevalence of such conditions are increasing worldwide, mainly due to population growth and poor lifestyle choices (1, 2). CVDs are the leading cause of mortality globally and a serious public health problem, primarily due to heart failure (HF) and myocardial infarction (MI) (3). The presence of atherosclerosis is the most common cause of both HF and MI (i.e., disruption of atherosclerotic plaques within an artery supplying the heart muscle can cause blockages that can lead to tissue death and HF). Besides acute or chronic coronary syndromes, atherosclerosis also participates in the development of cerebral ischemia or aneurysm (4). This knowledge together with the World Health Organization (WHO) reporting 31% of global deaths are CVD-related, of which 85% of deaths are due to heart attack and stroke (5), suggests atherosclerosis significantly impacts global mortality rates, but the full extent to which it contributes is not clear.

## Atherosclerosis and Associated Risk Factors

Atherosclerosis is a chronic inflammatory disease that occurs in large and medium-sized arteries (6). The layers of the coronary artery wall include the tunica adventitia, tunica media, and tunica intima. Among the various types of cells that constitute these layers, endothelial cells (ECs) in the intima layer and vascular smooth muscle cells (VSMCs) in the tunica media participate most in plaque formation, as well as macrophages (in particular M1 (pro-inflammatory) and M2 (anti-inflammatory) types), which also contribute (7, 8). Endothelial dysfunction, a critical point in atherogenesis, is characterized by a disturbed nitric oxide (NO) metabolism and reduced bioactivity of NO (9). Such dysfunction includes impaired NO synthesis and availability and imbalances in endothelium-synthesized relaxing and contracting factors such as angiotensin (Ang) and endothelin-1 (ET-1) (10). Recently a new relationship was established between hydrogen peroxide and Ang, which demonstrates the existence of a quality control process as part of the redox homeostatic mechanisms that occur within the vascular endothelium (11).

Atherosclerosis can occur when high blood pressure, high blood cholesterol, diabetes mellitus, cigarette smoking, or other factors damage the lining of the artery wall (12). These mechanical stimuli compromise the integrity of the endothelial barrier by activating signaling pathways that reduce the levels of NO (thereby inhibiting the migration and survival of endothelial cells) (13), and superoxide dismutase (SOD) (thereby increasing cellular oxidative stress) (14), which leads to the accumulation of apolipoprotein B (apoB)-containing lipoproteins [i.e., low-density lipoproteins (LDL)] (15), and ECs activation. The activation of ECs increases the production of reactive oxygen species (ROS) (16), which can oxidatively modify apoB and other lipoproteins (17, 18). The presence of such oxidized LDL (OxLDL) induces a pro-inflammatory environment and the deposition of lipids in the arterial wall (19). The deposition of lipoproteins in the arterial layers is often regarded as the first step in atherosclerosis development (20). Cholesterol filled lipoproteins influence both vascular and innate immune cells (ICs), and this interplay influences plaque formation and its properties (12, 21), whereby activated ECs induce a monocyte recruitment cascade that includes trans-endothelial migration (22). The monocytes then convert to pro-inflammatory macrophages (23), which internalize and degrade the lipoproteins in lysosomes before guiding the cholesterol component to the endoplasmic reticulum and its cholesteryl ester form into cytoplasmic lipid droplets to form foam cells (24, 25). Some amino acids (AAs) exert their proatherogenic and antiatherogenic effects by modulating macrophage cell activity and foam cell formation. Of the branched-chain amino acids (BCAAs), leucine (Leu) is the most extensively studied. A study using apolipoprotein E null (ApoE^−/−^) mice showed that Leu mediates its cardioprotective effects via improvement in lipid profiles and a decrease in systemic inflammation (26). These effects are likely related to the attenuation of foam cell formation mediated by a decrease in macrophage lipid content, dysregulated mitochondrial respiration, and ATP production. The vasoprotective roles of glycine (Gly) are partly mediated through the effects generated after binding to Gly-gated channels and chloride influx into macrophages, thus attenuating foam cell formation. In a study using New Zealand White rabbits, a decrease in macrophage accumulation and induction in macrophage apoptosis in intimal lesions were found to mediate the antiatherogenic effects of L-arginine (L-Arg) (27). Additionally, NO and L-citrulline are the products of the reaction between oxygen and L-Arg. NO is a potent vasodilative and antiatherogenic molecule, whilst methionine (Met), a sulfur-containing AA, exerts its proatherogenic effects through an increase in homocysteine (Hcy) production. Hyperhomocysteinemia promotes atherogenesis by foam cell production in addition to other mechanisms (28). During atherogenesis, a core comprised of such foam cells is covered with a fibrous cap produced by VSMCs that migrate into the intima (29); this process marks the transition from a fatty streak to a fibrous fatty lesion (30). With continued cholesterol accumulation, the fibrous fatty lesion eventually progresses into fibrotic plaques that can rupture and trigger major coronary events. Fortunately, large randomized trials with cholesterol-lowering drugs (31–35) report a reduction in total mortality, chronic heart disease deaths, and the risk of major coronary events from such interventions.

The connection between atherosclerosis and cholesterol accumulation is therefore, well-established (36). However, several recent studies indicate that the relative presence and/or absence of certain AAs may also contribute to atherosclerosis development and occurrence (37). One such study utilizing a rabbit atherosclerosis model reports alterations in the levels of several proteins, metabolites, and AAs in rabbit aorta, as well as in both rabbit and human plasma, suggesting that some of these molecules may be potential atherosclerosis biomarkers (38). Some specific AAs such as Gly and BCAAs such as valine (Val), Leu, and isoleucine (Ile) can ameliorate cell metabolic processes through mitochondrial biogenesis (39), influencing macrophage foam cells and altering lipid metabolism (37). The effect of the described amino acids on atherosclerosis and related diseases in animal models and humans is shown in Table 1. The chronically elevated levels of BCCAs and dysregulation of their catabolism can affect glucose metabolism through the suppression of the pyruvate dehydrogenase complex and lead to cardiac ischemic injury (41).

**Table 1:** Some effects of AAs observed in human and animal studies.

**Amino acid**	**Human studies**		**Animal studies**	
	**Study**	**Finding(s)**	**Experimental model**	**Finding(s)**
BCAAs	472 subjects (272 males and 200 females, age 42–97) Measurement of BCAA levels	Elevated level of BCAAs is positively and independently correlated with increased cIMT (40)	PP2Cm germ-line knockout mice Impaired BCAAs catabolism	BCAA chronic accumulation inhibits PDH activity, suppresses glucose metabolism, promotes ischemic cardiac injury (41)
Trp	13 healthy females; 12 males and 3 females subjected to surgical endoarteriectomy Measurement of Trp level	Lower serum level of Trp in patients with atheromatous plaques in comparison with healthy (42)	Male Sprague–Dawley rats Trp administration	Increased different serum AAs concentrations, decreased BCAAs, promoted the oxidation of fatty acid, reduced LDL level and fat deposition (43)
L-Arg	12 healthy older persons (age 73.8 ± 2.7) L-Arg supplementation	Improved artery diameter and endothelial function (44)	Hypercholesterol-emic male rabbits L-Arg supplementation	Improved endothelial function, reduction of atherosclerotic plaques (45)
L-Arg	20 males and 2 females (age 57± 9) with stable angina L-Arg supplementation	Improved exercise capacity (46)	Hypercholesterol-emic rabbits L-Arg supplementation	Improved NO-dependent vasodilator functioning, induced atheromatous lesion regression, and reversed endothelial dysfunction (47)
L-Arg	10 males and 10 females (age 59 ± 8) with CAD L-Arg administration	Vasodilated coronary arteries (48)	Hypercholesterol-emic rabbits L-Arg supplementation	Prevention of intimal thickening in coronary arteries, and increased macrophage accumulation in the intima layer (49)
h-Arg	282 heart failure patients (231 males and 51 females, age 55 ± 12) Measurement of h-Arg	Low plasma levels of h-Arg were associated with an increased fatal outcome risk from CVD and strokes (50)	C57BL/6J mice h-Arg supplementation	Protective effect in a post-myocardial infarction heart failure (51)
Tau	2,734 subjects (1,352 males and 1,382 females) Measurement of Tau	Inverse association between Tau levels and ischemic heart disease mortality (52)	New Zealand white rabbits Tau supplementation	Reduced myocardial apoptotic nuclei (53)
Tau	17 patients (11 males and 6 females) with congestive heart failure Tau supplementation	Improved systolic left ventricular functioning (54)	Male New Zealand white rabbits Tau supplementation	Decreased cholesterol, triglyceride, MDA and DC levels in the plasma, liver and aorta (55)
Tau	22 healthy males (age 18–29) Tau supplementation	Improved antioxidant effects, antagonism of Ang II action, and lipid profile (56)	Male homozygous apoE-deficient mice Tau supplementation	Reduced atherosclerotic lesion formation, decreased serum TBARS levels and oxidized LDL formation (57)
Cys	389 patients (242 males, 147 females, 41–65 years) with hyperlipidemia Measurement of Cys	Plasma Cys levels being significantly lower in healthy individuals than in carotid atherosclerosis in symptomatic patients (58)	?	?
Gly	80,003 participants (meta-analysis) Identification of Gly genetic loci	Genetically associated with lower CHD risk (identified 27 genetic loci) (59)	Male Wistar rats Gly supplementation	Reduced ${\text{O}}_{2}^{-}$, protein carbonyl and lipid peroxidation, increased eNOS, NO and biosynthesis of glutathione in the aorta (60)
Gly	4,150 patients (72% men; median age 62 years) Measurement of DMG	DMG plasma levels were associated with the risk of AMI in patients with stable angina pectoris (61)	Female Sprague-Dawley rats Gly supplementation	Prevented aggregation of platelets, increased bleeding time (62)

*AAs, amino acids; AMI, acute myocardial infarction; Ang II, angiotensin II; apoE, apolipoprotein E; BCAAs, branched-chain amino acids; CAD, coronary artery disease; CatC, cathepsin C; cIMT, carotid intima-media thickness; CHD, chronic heart disease; CVD, cardiovascular disease; Cys, cysteine; DC, diene conjugate; DMG, dimethylglycine; eNOS, endothelial nitric oxide synthase; Gly, glycine; h-Arg, homoarginine; L-Arg, L arginine; LDL, low-density lipoprotein; LDLR^−/−^, low-density lipoprotein receptor null allele mice; MDA, malondialdehyde; NO, nitric oxide; PDH, pyruvate dehydrogenase; Tau, taurine; TBARS, thiobarbituric acid-reactive substances; Trp, tryptophan. The “?” sign indicates a conclusion could not be reached with the currently available data*.

Thus, BCAAs may also impact atherosclerosis prevention and offer a novel therapeutic strategy for certain individuals at risk of coronary events (37). ICs are impaired during atherosclerotic formation, and such aberrations of normal functioning correlate with the levels of specific AAs. AAs are fundamental elements for protein metabolism and participate in different cellular mechanisms for energy generation (63). Other AAs are indirectly associated with atherosclerosis as they play essential roles in vascular functioning; for example, L-Arg is involved in NO synthesis (64, 65). On the other hand, despite such essential roles for some AAs, elevated levels of BCAAs can lead to pathological conditions, including neurological disorders and cardiomyopathy (66). In this review, we summarize the recent knowledge related to the role of AAs, including the BCAAs, Tryptophan (Trp), L-Arg, Taurine (Tau), Cysteine (Cys), Homoarginine (h-Arg) and Gly in the development and progression of atherosclerosis and atherosclerosis-related CVDs.

## Essential AAs and Atherosclerosis

### BCAAs Catabolism

**Figure 1:**
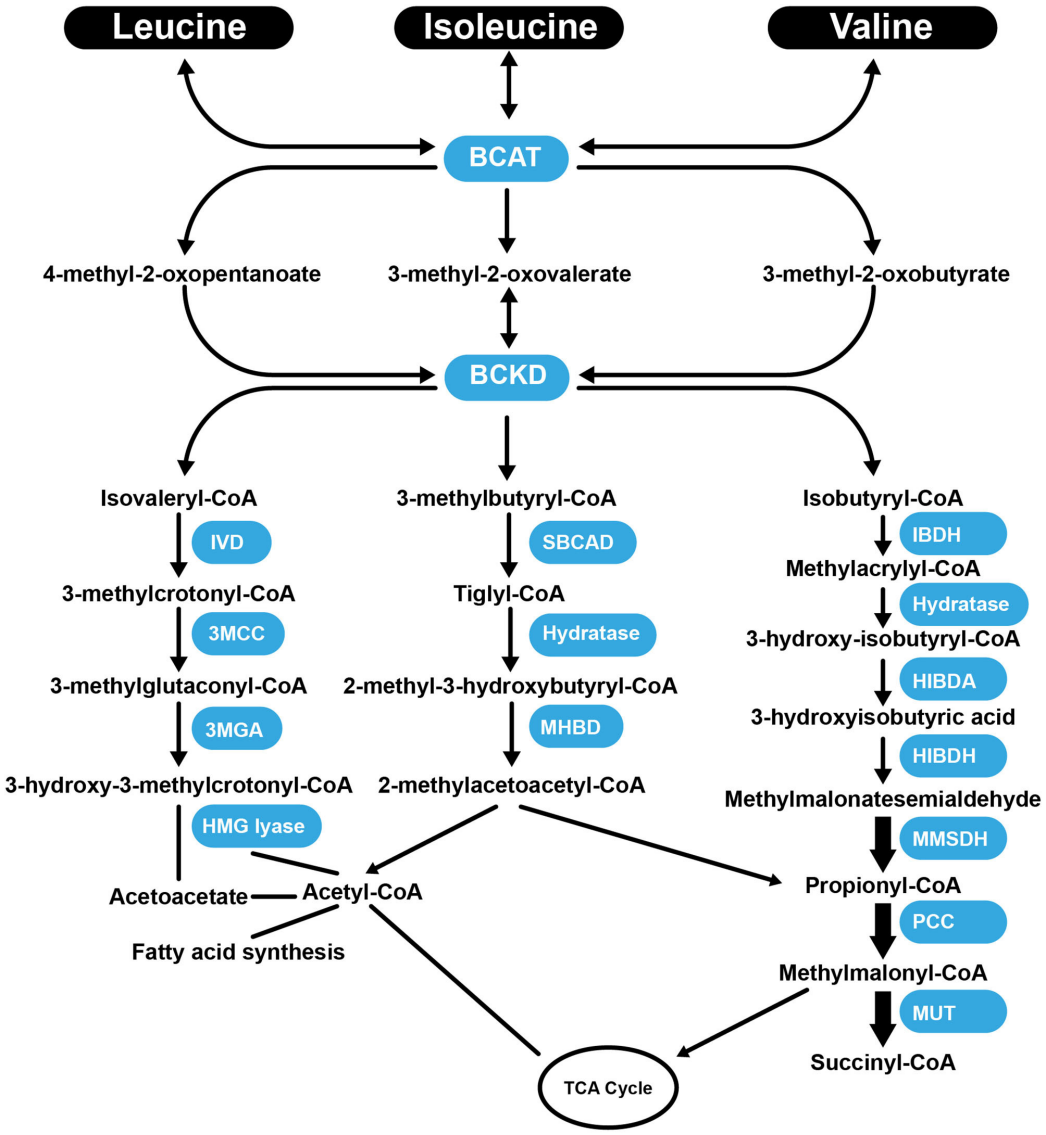
Summary of the catabolism pathways of BCAAs. BCAT, branched-chain amino acid aminotransferase; BCKD, Branched-chain α-ketoacid dehydrogenas; IVD, isovaleryl-CoA dehydrogenase; 3MCC, 3-methylcrotonyl-CoA carboxylase, 3MGA, 3-methylglutaconic-CoA hydratase; HMG lyase, 3-hydroxy-3-methylgutaryl-CoA lyase; CBCAD, methylbutyryl CoA dehydrogenase; MHBD, 2-methyl-3-hydroxyisobutyric dehydrogenase; IBDH, isobutyryl-CoA-methyl-3-hydroxyisobutyric dehydrogenase; HIBDA, 3-hydroxyisobutyryl-CoA deacylase; HIBDH, 3-hydroxyisobutyrate dehydrogenase; MMSDH, methylmalonic semialdehyde dehydrogenase; PCC, propionyl-CoA carboxylase; MUT, methylmalonyl-CoA mutase; TCA Cycle, tricaboxylic acid cycle.

Val, Leu, and Ile are essential BCAAs vital for the growth and functioning of cells and organs. They differ in their side-chain properties (e.g., hydrophobicity and conformation). The BCAAs constitute nearly 40% of all the AAs in the body (67). They represent about 35% of the essential AAs found in mammalian proteins (68). Leu stimulates myofibrillar protein synthesis (69, 70). In the presence of lowered glucose, Leu, and its non-metabolizable analog, 2-aminobicyclo (2, 2, 1) heptane-2-carboxylic acid stimulate insulin secretion from the β cells of the pancreas (71). There is a strong requirement of Leu during the synthesis of sterols in adipose and muscle tissue (72). Approximately 60% of Leu is metabolized after several hours, of which about 5% is converted to β-hydroxy β-methylbutyric acid (73), and 40% is converted to acetyl-coenzyme A (CoA) (74), which is used for the synthesis of other compounds. Catabolism of Leu is controlled by the enzymes, branched-chain amino acid aminotransferase (BCAT), and branched-chain α-ketoacid dehydrogenase (BCKD) to produce isovaleryl-CoA (75). Isovaleryl-CoA is further processed by the isovaleryl-CoA dehydrogenase and converted to 3-methylcrotonyl-CoA, which is used in the synthesis of acetyl-CoA and acetoacetate by the enzymes 3-methylcrotonyl-CoA carboxylase, 3-methylglutaconic-CoA hydratase and 3-hydroxy-3-methylglutaryl-CoA lyase (Figure 1). Following transamination, Ile is converted into tiglyl-CoA, 2-methyl-3-hydroxybutyryl-CoA and 2-methylacetoacetyl-CoA. The latter is further converted to propionyl-CoA (a glucogenic precursor) and acetyl-CoA, which enter into the tricarboxylic acid cycle (Figure 1). Familial deficiency of the 2-methyl-3-hydroxybutyryl-CoA dehydrogenase enzyme causes progressive infantile neurodegeneration (76). Catabolism of Val starts with the transamination process, which is catalyzed by BCAT, which then through the action of BCKD produces isobutyryl-CoA, which is successively converted into propionyl-CoA and methylmalonyl-CoA. The latter compound enters into the tricarboxylic acid cycle (77) (Figure 1). Val decomposition products may act as signaling molecules. The only metabolite of BCAAs that does not have a covalent attachment to CoA is 3-hydroxyisobutyric acid (3-HIB), which can easily leave the mitochondrial matrix. The 3-HIB metabolite is synthesized within muscle; the surrounding plasma concentrations of 3-HIB typically range between 30 and 50 μM (78). Secreted 3-HIB acts as a paracrine signal acting on surrounding microvascular ECs, where it promotes the uptake of fatty acids and lipid accumulation in the muscle and can contribute to the development of insulin resistance (IR) in mice (78).

### The Role of BCAAs in Atherosclerosis

Alterations in the levels of BCAAs are associated with disorders, including renal failure, atherosclerosis, and cancer (79). Specifically, after circulating BCAAs were demonstrated to correlate with increased risk for cardiovascular events and IR, Mels *et al*. assessed whether BCAA levels are associated with carotid intima-media thickness (cIMT) under hyperglycaemic conditions (80). Using regression analyses, they determined that an independent correlation exists between BCAAs and cIMT in individuals with high HbA1c levels and suggested that cardiovascular deterioration possibly accompanies hyperglycemia (80). Also, Yang *et al*. investigated whether AAs could be used to identify subclinical atherosclerosis subjects at risk of developing coronary artery disease (CAD) caused by atherosclerosis, as BCAA levels had previously been shown to correlate with atherogenic dyslipidemia (40). Based on carotid artery images and BCAA serum levels, they determined a significant and independent positive correlation between BCAAs (Val, Ile, and Leu) and increased cIMT (40). Identifying such risk factors offers hope for the prevention of early atherosclerosis (40, 81). However, other studies have revealed that dietary and circulating Ile, Leu, and Val levels are not correlated, suggesting that other factors beyond dietary intake influence the levels of BCAAs in plasma (82, 83). The most likely explanation is that alterations in the metabolism of BCAAs contribute significantly to the elevated levels of circulating BCAAs observed, rather than changes in BCAA intake (82, 83). Several studies have shown an association between BCAAs, their metabolites, and CAD (84). For example, mass spectrometric analysis of plasma samples taken from almost 2,000 CVD patients showed 63 differentially synthesized metabolites and further suggested that the levels of some BCAAs and their metabolites are related to the severity of CAD (84). The increase in plasma BCAA levels in CAD may be due to lower BCAA uptake and usage by muscles and other tissue (85), or lowered activity of the critical enzyme BCKD complex (BCKDC) responsible for the degradation of all BCAAs in animals (86, 87). The accumulation of BCKDC has been linked to heart failure via direct perturbation of respiration and increased ROS synthesis within the mitochondria (88). Defects in BCAA catabolism can more widely affect metabolism in murine failing hearts through alterations in Krupel-like factor-15 (89). Supplementation with BCAAs can decrease heart muscle injury and ameliorate hematological parameters in rats during endurance exercises (90). Contrary to this, several prospective metabolomic studies of incident coronary heart disease (CHD), CAD, and MI failed to identify associations of any BCAAs with CVD (91–93). Supplementation with BCAAs at a dose of 1.5 mg/g body weight/day in drinking water preserves skeletal muscle weight and ameliorates HF in a rat model (94). Also, Leu (1.5% w/v) supplementation in ApoE^−/−^ mice reduces the growth of atherosclerotic plaques via reduced inflammation and an improvement in the lipid profile (increased high-density lipoprotein cholesterol (HDLc) and reduced LDL) (26). Leu (1.3% dietary protein-energy intake) was inversely associated with arterial stiffness and atherosclerosis in females (95). Leu supplementation in humans, at a dose of 5 g/day for 3 weeks, modifies lipid metabolism in macrophages while simultaneously enhancing mitochondrial respiration, which may offer a potential strategy to attenuate atherosclerosis development in humans (96). Regarding cardiovascular risk, there are positive and negative atherogenic roles of BCAAs. Certain BCAAs, in particular Leu, are well-known for their attenuative effects on macrophage lipid accumulation and subsequent formation of foam cells in blood vessel walls. This role is related to the decrease in cholesterol and triglyceride macrophage content. A decrease in cholesterol content is obtained through reduced very-low-density lipoprotein (VLDL) uptake, inhibition of cholesterol synthesis, and cholesterol cell efflux. On the other hand, a decrease in cellular triglycerides results from inhibition of the enzyme, diacylglycerol acyltransferase-1, which catalyzes the synthesis of triglycerides in macrophages. One particular study observed increased levels of circulating BCAAs in CVD, diabetes, IR, obesity, and healthy individuals, independently of body mass index (97). Metabolic status is the key element that determines whether increased levels of a particular BCAA has a positive or negative influence on CVD risk (97, 98). In this regard, it is known that some stimuli can impact tissues involved in BCAA clearance, such as brown adipose tissue (BAT), a well-known thermogenic (and BCAA catabolic) organ. Cold exposure influences BAT to promote the oxidation of BCAAs to obtain enough energy for thermogenesis produced by mitochondria, thus promoting systemic BCAA clearance. Cold exposure additionally affects lipid catabolism but does not influence glycaemia. Brown adipocytes take up and transport BCAAs via mitochondrial BCAT1 and Solute Carrier Family 25 Member 44 gene (SLC25A44). Defects in these processes have been shown to impair the thermogenesis and influence BCAA systemic clearance leading to obesity and conditions linked to IR (98). Collectively, BCAAs appear to represent important indicators of CVD risk, and further work is required to fully understand their involvement in such pathologies.

### Trp Catabolism

**Figure 2:**
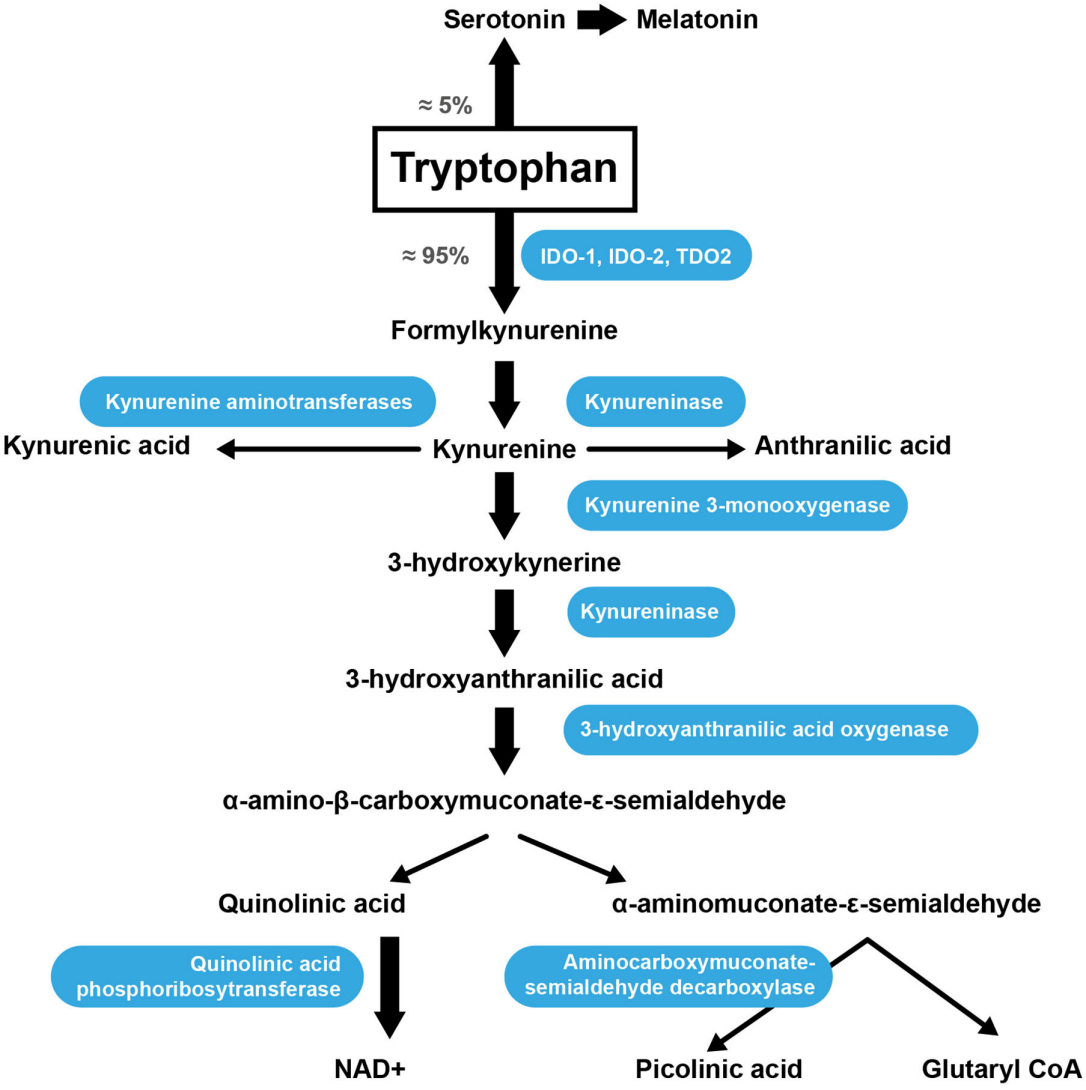
Trp catabolism via the KP. IDO-1, indoleamine 2,3-dioxygenase; IDO-2, indoleamine 2,3-dioxygenase 2; TDO2, tryptophan 2,3-dioxygenase.

Trp is an AA acquired through the diet because the human body is unable to synthesize it. Once absorbed through the intestine into theportal blood, 85–90% of Trp is transported in the plasma bound to serum albumin, while only 10–15% of the total plasma Trp is in the free form (99). The metabolic pathway responsible for approximately 90% of Trp catabolism is the kynurenine synthesis pathway (KP) (Figure 2) (100). Trp is oxidized by cleavage of the indole ring, which is initiated either by indoleamine 2,3-dioxygenase 1 (IDO-1), indoleamine 2,3-dioxygenase 2 (IDO-2) or tryptophan 2,3-dioxygenase (TDO2) (101). The TDO2 enzyme can be induced by the presence of Trp or by corticosteroids. IDO-1 is the predominant enzyme found in most cell types and is stimulated by inflammatory molecules (102). The KP produces several intermediate metabolites, of which kynurenine (KYN) is the first stable intermediate formed (103). Subsequently, other neuroactive intermediates are generated, including 3-hydroxyanthranilic acid (HAA) (104), quinolinic acid (105), and picolinic acid. Picolinic acid is a natural iron and zinc (Zn) chelator and serves as an endogenous neuroprotectant (106). Serotonin [5-hydroxytryptamine (5-HT)] synthesis is one of the most important Trp pathways (107). Note that 3% of dietary Trp is used for 5-HT synthesis throughout the body, while only 1% of dietary Trp is used for 5-HT synthesis in the brain (108), where Trp is primarily involved in melatonin synthesis (Figure 2) (109).

### Role of Trp in Atherosclerosis

It has been proposed that IDO-1 impacts atherosclerotic processes via multiple mechanisms. Cole *et al*. found that IDO-1 is protective against atherosclerosis and that the downstream products that occur following the breakdown of Trp create a feedback loop that controls athero-inflammation and atherogenesis (110). Also, inhibition of IDO-1 in ApoE^−/−^ mice has been shown to lead to increased vascular inflammation and atherosclerosis. Zhang *et al*. found that the downstream metabolite, HAA lowers plasma lipids (including cholesterol and triglycerides) and decreases atherosclerosis in hypercholesterolemic mice (111). Another suggested mechanism by which IDO-1 protects against atherosclerosis is through inhibition of IL-10 production by a kynurenic acid (KA)-induced mechanism (112).

A study examining patients with CHD found that they had a low serum Trp and an elevated serum KYN/Trp ratio (113). The sera of patients with atheromatous plaques was also found to have lower levels of Trp compared with healthy controls (42). After oral supplementation with L-Trp, patients suffering from MI or angina pectoris showed lower Trp and higher KYN concentrations in serum than healthy controls (114). Elevated concentrations of KA, a catabolite of Trp, in atheromatous plaques are associated with unstable human atherosclerotic lesions, whereas lower levels of KA are found in stable fibrous plaques (112). Serum Trp and KYN levels were also estimated by a chromatography-based method in subjects undergoing coronary angiography and in persons with healthy coronary arteries; following L-Trp supplementation, lower serum Trp and higher KYN concentrations were detected in patients suffering from MI or angina pectoris compared with healthy controls (113). Reduced plasma Trp levels are associated with various disease states. Further work is required to understand whether Trp supplementation may represent a therapeutic (or preventative) strategy to improve cardiovascular health. The role of IDO in vascular biology warrants further investigation.

## Conditionally Essential AAs in Atherosclerosis

Of the classically categorized non-essential AAs, some have been shown to become essential under specific conditions such as illness, injury, or stress. Thus, they can be classified as conditionally essential. The known conditionally essential AAs include Arg, Cys, Gly, glutamine, proline, tyrosine, and Tau. Several conditionally essential AAs play a role in atherosclerosis, including L-Arg, Tau, Cys, and Gly (115).

### L-Arg Metabolism

L-Arg is a conditionally essential AA, as our ability to synthesize L-Arg is altered at different developmental stages and in certain disease states (116). L-Arg is essential for wound healing (117), biosynthesis of NO (118), and blood pressure regulation (119). Free L-Arg is obtained by *de novo* synthesis, from the diet or during protein turnover. Synthesis of L-Arg in humans occurs in the epithelial cells of the small intestine and kidney (120). Impaired renal function may decrease L-Arg synthesis, increasing the dietary requirement (121).

L-Arg is synthesized from the non-essential AA, citrulline by the successive action of the cytosolic enzymes argininosuccinate synthetase and argininosuccinate lyase (122). L-Arg can also be synthesized from glutamine in the gut (123) and citrulline through the condensation of ornithine and carbamoyl phosphate in the small bowel (124, 125). Citrulline is also produced from L-Arg through the enzymatic activity of NO synthase (NOS) and from asymmetric dimethylarginine (ADMA) through dimethylarginine dimethylaminohydrolase (DDAH) (126). ADMA, an endogenous L-Arg analog, inhibits NOS activity and, consequently, the production of NO. The dimethylarginine molecules can be converted into L-Arg by DDAH (127). NO produced by inducible NOS (iNOS) produces peroxynitrite radicals, which promotes atherogenesis (128).

**Figure 3:**
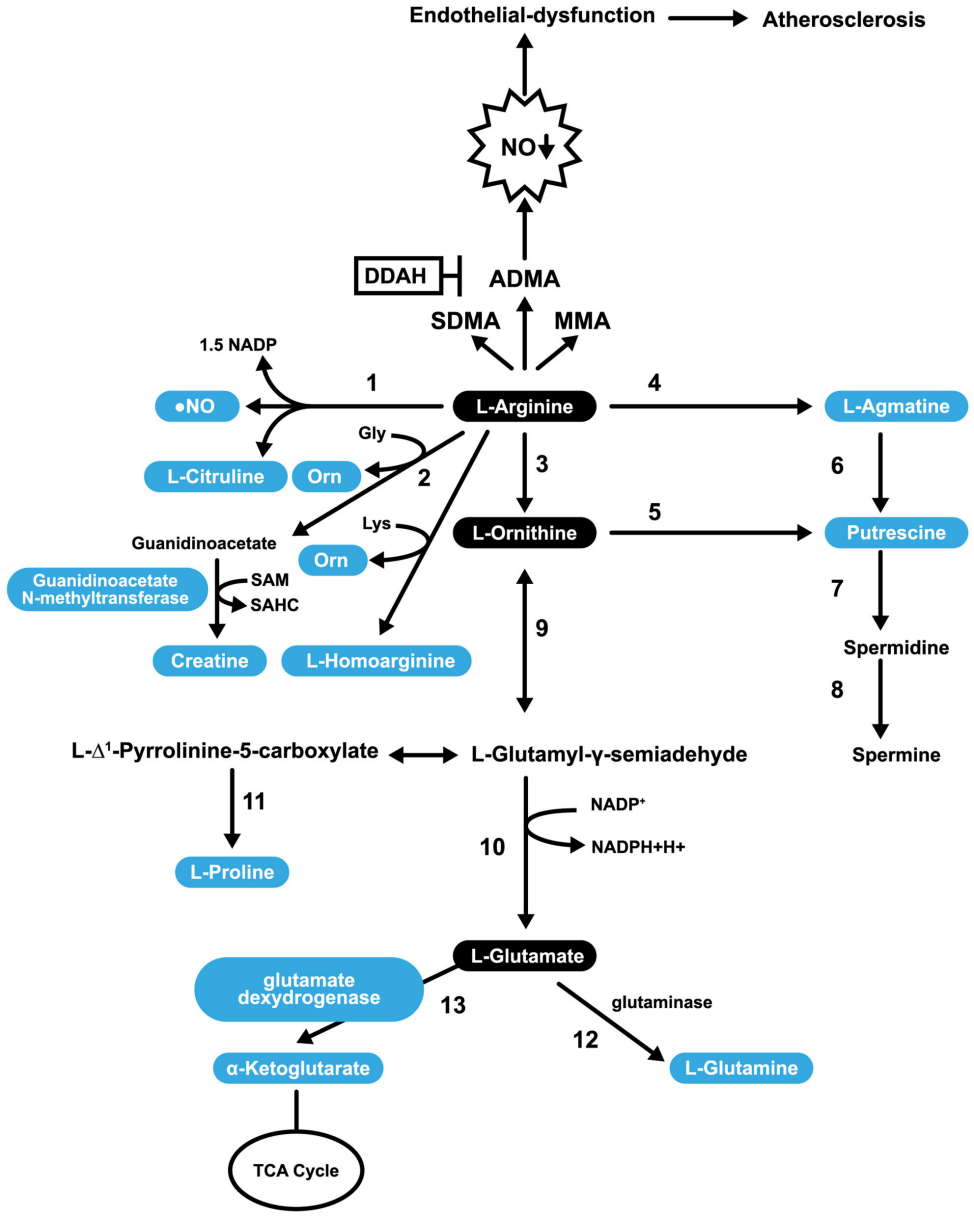
Pathways involved in L-Arg Catabolism. 1, NO synthases; 2, arginine: glycine amidinotransferase (AGAT); 3, arginase; 4, arginine decarboxylase; 5, ornithine decarboxylase; 6, agmatinase; 7, spermidine synthase; 8, spermine synthase; 9, ornithine aminotransferase; 10, pyrroline-5-carboxylate dehydrogenase; 11, pyrroline-5-carboxylate reductase; 12, glutaminase; 13, glutamate dexydrogenase.

Catabolism of L-Arg occurs through the action of several enzymes in mammalian cells; most notably, NOS, arginine glycine amidinotransferase (AGAT), arginase 1 (ARG1), arginase 2 (ARG2) and arginine decarboxylase. There are cytosolic and mitochondrial isoforms of ARG (126). ARG catalyzes the final step in the urea cycle (conversion of L-Arg into L-ornithine; Figure 3). The enzyme arginine decarboxylase utilizes L-Arg to produce agmatine (AG). AG has a broad spectrum of actions (on neurotransmitter systems, atherosclerosis, NO synthesis, and polyamine metabolism), suggesting the potential for its supplementation as a strategy to treat various disorders (129, 130). The enzyme AGAT utilizes L-Arg and Gly to produce guanidinoacetate, which is further metabolized to form creatine. This enzyme can also utilize lysine as a substrate to synthesize h-Arg (which is discussed in more detail in section h-Arg Metabolism and Atherosclerosis) and ornithine.

L-Arg is a substrate for the synthesis of putrescine (PUTR), which can be made in two ways. In the first, L-Arg is transformed into AG (by arginine decarboxylase), then agmatinase converts AG into PUTR. In the second mechanism, L-Arg is converted into L-ornithine, and L-ornithine is converted into PUTR. In both cases, PUTR is further converted, by the action of spermidine synthase and spermine synthase, into spermine (Figure 3). PUTR is a polyamine growth factor necessary for cell division. PUTR is found in semen, as is spermidine and spermine (131). PUTR has a role in continual and controlled efferocytosis, which is the process by which macrophages clear apoptotic cells. Yurdagul *et al*. found that this process is enhanced in apoptotic cells by the conversion of Arg and ornithine to PUTR that is catalyzed by the macrophage enzymes, ARG, and ornithine decarboxylase. The role of PUTR is to facilitate subsequent rounds of dead cell internalization. Impaired efferocytosis is seen in chronic diseases and is associated with atherosclerosis's acceleration, a consequence of the inhibition of apoptosis. The net result is inflammation and necrosis of non-internalized apoptotic cells. Such inflammation transforms stable atherosclerotic plaques into unstable forms that can rupture and cause embolization (132).

L-Arg can be converted into glutamate (shown in Figure 3) and can undergo several post-translational modifications. For example, specific L-Arg residues in proteins can be methylated by a family of protein arginine methyltransferases resulting in transformations to either monomethyl arginine or symmetric dimethylarginine or ADMA. ADMA inhibits constitutive endothelial and neuronal NOS (eNOS and nNOS) (133) and is a less potent inhibitor of iNOS (134). After the degradation of proteins, methylated L-Arg residues are released into a free AA pool where they competitively inhibit NOS activity (135). In addition to methylation, L-Arg can also be ribosylated, citrullinated, or phosphorylated (136). A healthy diet provides ~5.4 g of L-Arg per day (137). Only 40% of our L-Arg is taken up directly from the diet through the portal blood, with the rest sourced through the action of intestinal arginase (138). Impairment of the L-Arg/eNOS pathway is implicated in CAD (139). L-Arg may play a role in the prevention of CAD through its capacity to stimulate the pathway mentioned above, and some NO-independent actions.

### Role of L-Arg in Atherosclerosis

NO produced by L-Arg possesses anti-atherogenic properties and plays a role in cardiovascular protection. However, in the pathological state, eNOS overactivation causes over-production of NO, leading to the development of atherosclerosis and damage of ECs (140). Modern therapies used to treat atherosclerosis often work to increase the bioavailability of NO in the vascular endothelium.

In a L-Arg supplementation study (2.25% final concentration in drinking water) in experimental hypercholesterolemic male rabbits, an improvement in endothelial function, a reduction of atherosclerotic plaques and the consequent reduction of atherosclerosis were reported (45). In rabbits, administration of L-Arg (2.25% in drinking water) in combination with the NO synthesis inhibitor, N-nitroarginine methyl ester, led to partially renewed endothelium-dependent relaxation of NO production (as indicated by increased NO^3−^ excretion) and restored endothelial function in hypercholesterolemia (as indicated by enhanced NO production and reduced early breakdown of NO by O_2_) (141). In hypercholesterolemic rabbits, L-Arg (2.0% in drinking water) also improved NO-dependent vasodilator functioning, induced atheromatous lesion regression and reversed endothelial dysfunction. The mechanisms thought to be responsible for this effect include the restoration of NO synthase substrate availability and the reduction of vascular oxidative stress (47). Hypercholesterolemic rabbits were also used to show that supplementation with L-Arg (2.25% in the drinking water) prevents intimal thickening in coronary arteries and significantly decreases monocyte and macrophage accumulation in the intima layer for a period extending from 1 to 10 weeks (49). In hypercholesterolemic rabbits, oral L-Arg (2.5% in the drinking water) has also been shown to diminish the formation of atherosclerotic lesions (142). Further, in hypercholesterolemic rabbits, L-Arg (2% in drinking water) or alpha-tocopherol (300 mg/day) improves endothelium-dependent vasodilator function, NO production (143), and reduces oxidative stress and progression of atherosclerosis in experimental animals. Supplementation with L-Arg improves endothelial functioning in hypercholesterolemic young adults without changing the lipid profile, demonstrating that this AA has anti-atherogenic properties (144). In humans, aging is an independent risk factor for atherosclerosis development (145); elderly patients with CVDs exhibit flow-mediated dilation of the brachial artery. Further to this, a prospective randomized crossover trial wherein 12 healthy older persons (aged 73.8 ± 2.7 years) were administered 8 g of L-Arg twice daily for 14 days (44). It was reported that artery diameter measured by high-resolution ultrasound, significantly improved after L-Arg supplementation, as did endothelial function, probably due to a normalization of the L-Arg/ADMA ratio (44). Ceremuzynski *et al*. reported that CAD patients with stable angina exhibit an improved exercise capacity after being supplemented with 6 g of L-Arg daily for 3 days (46). L-Arg at doses of 50 and 150 mmol/min via intracoronary infusions for 8 min also led to vasodilation of coronary arteries in CAD patients (48). Another study additionally used intravascular ultrasonography to measure neointimal volume in stents after L-Arg treatment (100 mg/ml) through the catheter for 15 min; a 35% increase in neointimal volume was measured after L-Arg treatment (compared with placebo-treated patients), however, luminal volume remained the same (146). A study by Creager *et al*. that included 14 hypercholesterolemic individuals found that the intravenous administration of L-Arg (administered at a rate of 10 mg/kg per min) improves endothelium-dependent vasodilation (147).

Contrary to these findings, Blum *et al*. reported that CAD patients on an appropriate therapy that were additionally administered L-Arg (9 g supplementation every day for 1 month), showed a significantly increased level of L-Arg in plasma, yet NO bioavailability did not improve (148). Similarly, in a study involving male subjects that were followed up over 10 years, it was found that increased dietary L-Arg intake did not lower the risk of CHD mortality (149).

In a double-blind, randomized study, subjects administered 3 g of L-Arg three times a day for 6 months exhibited increased coronary blood flow in response to increased acetylcholine, which was explained by a decrease in the levels of plasma endothelin in the L-Arg-treated group compared with the placebo group (150). Results from this study support the concept that L-Arg is a suitable therapeutic agent for patients suffering from coronary endothelial dysfunction and non-obstructive CAD (cases in which atherosclerotic plaques are not expected to obstruct blood flow or cause anginal symptoms). In a second double-blind, randomized crossover study, ten male patients with coronary atherosclerosis were either administered with 7 g of L-Arg three times per day or placebo for 3 days, with a washout period of 10 days. Elevated levels of plasma L-Arg and endothelium-dependent dilatation were observed in the patients administered L-Arg. This suggests L-Arg improves the dilatation of the brachial artery and reduces the adhesion of monocytes/ECs (151). In another study, CAD patients were intravenously administered L-Arg (5 mg/kg/min for 20 min) yet showed no improvement in ST-segment responses or exercise tolerance, despite the ability of acetylcholine to improve forearm vasomotor responses (152). L-Arg given at a dose of either 2.5 g/m^2^ or 5 g/m^2^ three times a day was also unable to improve endothelial function in chronic renal failure patients (153). In atherosclerotic diseases, such as peripheral arterial occlusive disease and CAD, long-term L-Arg supplementation (10 g/day for 3 or 6 months) may lead to better utilization of nitrite by improving the kidney reabsorption of carbonic anhydrase-dependent nitrite. However, it is important to point out that the optimum dose of oral L-Arg remains undetermined for diseases with NO-related dysfunction (154). Some studies in patients with atherosclerosis and related complications show benefits with L-Arg administration and others not. There are several possible explanations:

1. One possibility may be variations between patients (demographic characteristics, age, gender). Also, parameters like the optimal dose and duration of L-Arg treatment are not standardized.
2. L-Arg supplementation does not always enhance NO synthesis because guanidine-methylated L-Arg derivatives that inhibit NOS activity are also sources from L-Arg. Simultaneous generation of methylated L-Arg residues from supplemented L-Arg may negate the positive effects of L-Arg on NO. In healthy individuals, ADMA infusion causes a temporary reduction in the cardiac output and induces a reduction of effective renal plasma flow in a dose-dependent manner (155).
3. ARG activity may influence L-Arg's cellular levels, decreasing NO production resulting in endothelial dysfunction (156).
4. A large proportion of L-Arg flux is used to synthesize creatine via the enzyme AGAT (157). The creatine generation is estimated to consume ~70% of labile methyl groups, with S-adenosyl methionine (SAM) serving as the methyl donor (Figure 3). When the methyl groups are donated to guanidinoacetate, SAM is converted to S-adenosyl homocysteine and, eventually, to Hcy. Since Hcy has adverse effects on endothelial function, L-Arg supplementation can also perturb endothelial function (158, 159).
5. L-Arg supplementation has a beneficial effect in hypercholesterolemia, probably because OxLDL and lysophosphatidylcholine are unable to inhibit protein transport (160). L-Arg competes with cationic AAs for cell uptake via the y+ AA transporter system, and increased L-Arg supplementation may increase L-Arg concentration in the cells by competitively inhibiting cellular uptake of other cationic AA. This relates to the Arg paradox, a phenomenon where exogenous L-Arg leads to NO-mediated biological effects even though NOS, which utilizes L-Arg as a substrate, is saturated (161).
6. L-Arg can undergo a decarboxylation reaction to produce AG, a competitive NOS inhibitor (162).

More studies are required to identify which individuals would benefit from L-Arg and those in which it should be used with caution (if at all). The optimum oral dosage of L-Arg is also not established in these and other diseases associated with NO-related dysfunction.

### h-Arg Metabolism and Atherosclerosis

h-Arg is a non-essential cationic AA that is synthesized during the catabolism of lysine or the transamination of L-Arg (Figure 3). These reactions involve ornithine transcarbamoylase, an enzyme from the urea cycle, or AGAT, an enzyme from the creatine biosynthesis pathway. h-Arg is found in the liver, kidneys, brain, and small intestine of humans and animals (163). h-Arg is an Arg homolog, that possesses an additional methylene group in its main chain and may represent a major NOS substrate (163). ARG also has the potential to alter h-Arg levels (164). Non-essential AAs can be synthesized in humans (and other animals) in addition to being obtained from the diet. The primary substrate for their synthesis is glucose (165). The plasma h-Arg concentration is positively associated with endothelial functioning (166), a reduction in platelet aggregation (167), and stimulation of insulin secretion (168). A study that performed angiographies on over 3,000 patients with acute coronary syndrome revealed that the group of patients with the highest plasma concentration of h-Arg had the most favorable outcomes (169), suggesting a protective effect in this context. Similarly, results of a separate study with type 2 diabetes mellitus patients undergoing hemodialysis for <2 years, revealed that lower h-Arg levels associate with negative outcomes including a lower estimated glomerular filtration rate, low plasma serum albumin, low body mass index and lower levels of LDL cholesterol (170). The study also found that patients on hemodialysis had lower plasma h-Arg levels and experienced significantly higher mortality rates (170). Low serum levels of h-Arg are associated with an increased fatal outcome risk from CVD and strokes (50). A Finnish longitudinal study involving over 4,000 children and adolescents that focused on the evolution of cardiovascular risk factors from childhood, revealed that plasma h-Arg levels are directly associated with cardiometabolic disease risk and that low levels predict increased mortality risk (171). In the Hoorn study, it was found that h-Arg levels are higher in older men than in women and that low h-Arg plasma concentrations are significantly associated with all-cause mortality and, in particular, with cardiovascular mortality (172). A prospective observational study that included patients with intermittent claudication reported h-Arg/ADMA and h-Arg/symmetric dimethylarginine ratios to be independent predictors for events in patients with lower extremity arterial disease (LEAD, or atherosclerotic stenosis) and long-term cardiovascular mortality (173). Studies utilizing mouse models have demonstrated NOS and arginase can metabolize h-Arg (100). Supplementation with 14 mg/L h-Arg hydrochloride in the drinking water for 4 weeks has a direct protective effect in a post-myocardial infarction HF murine model (51). Mice treated with h-Arg (at 30 mg/kg/day in saline) showed reduced neointima hyperplasia in balloon-injured rat carotid arteries compared with supplemented mice (174). The weight of evidence suggests that h-Arg may be more than just a marker of the adverse outcomes, but play a direct protective role in cardiovascular and metabolic pathologies (175). Additional clinical studies in humans involving h-Arg are needed to confirm its beneficial effects in atherosclerosis and related disorders.

### Tau Metabolism

**Figure 4:**
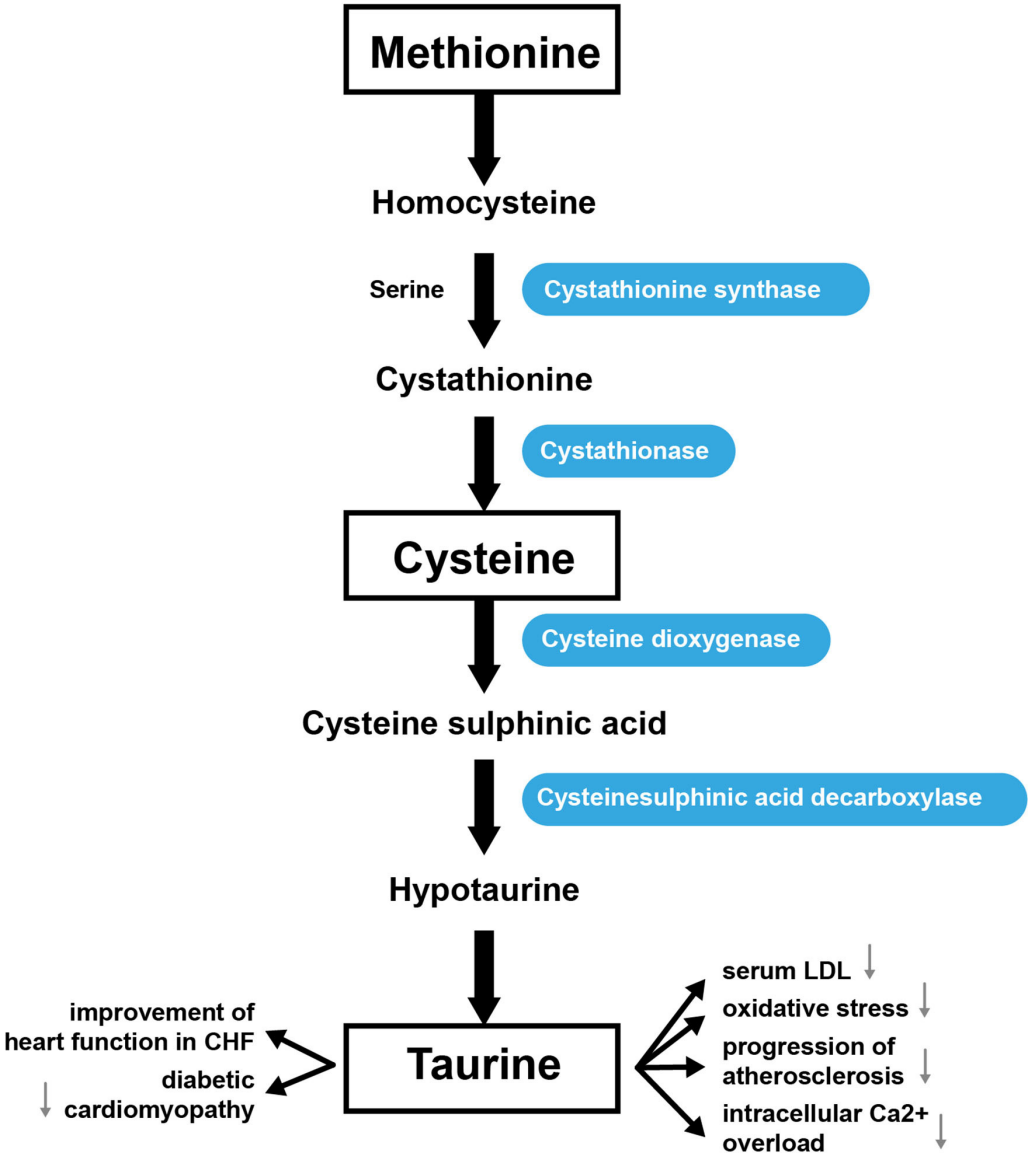
General pathway of Tau synthesis from Met.

Tau (2-aminoethanesulfonic acid) is the most prevalent intracellular sulfur-containing neutral β-amino acid (176). Tau is non-essential in rodents, but essential in cats (177) and may be conditionally essential in humans (178, 179). Tau can be obtained through the diet or through biosynthesis utilizing sulfur-containing AAs, such as Cys and Met (180). Cysteine dioxygenase converts Cys into cysteine sufinic acid. Then cysteine sulfinic acid decarboxylase converts cysteine sulfinic acid to hypotaurine, which can then be modified to generate Tau (181). Met can also be metabolized to form Cys for Tau synthesis. For this, Met is converted into Hcy, which cystathione synthase converts into cystathionine. Finally, cystathionine is then converted into Cys by cystathionase (182). Tau makes up ~0.1% of the body's total weight (180). The highest concentrations of Tau are usually found in the heart, brain, and musculature; it is less abundant in plasma. In the heart, Tau represents up to 60% of the total free AA pool. Concentrations of Tau in different species range from 3.5 μmol/g wet weight in cows to 30 μmol/g in rats (183). Tau is synthesized mainly in the liver or in the brain from Cys or Met, as indicated above (Figure 4) (184). The kidneys regulate the total body pool of Tau by regulating its tubular reabsorption (185). Tau has a cytoprotective role via neutralization of hypochlorous acid (186), and diminishes the synthesis of free radicals in mitochondria (187). Tau also regulates cellular functions such as the cell cycle, cell death/survival, and Tau depletion stimulates unfolding protein response (188). Tau regulates the expression of enzymes involved in BCAA catabolism (including BCAT 2; mitochondrial, BCAT 1, cytosolic; branched-chain keto acid dehydrogenase; 3-hydroxy-3-methylglutaryl-CoA lyase) (189).

Tau is essential for maintaining an osmotic balance (in response to high osmotic load, intracellular Tau increases) (190), normal retinal ganglion cell survival (191), and has an antiarrhythmic action (192). Tau supplementation has been approved for the treatment of congestive heart failure in Japan (54) and has been shown to induce regression of serum cholesterol levels in atherogenic animal models (193), as it inhibits cholesterol synthesis (194). Tau deficiency impairs the contractile function of both cardiac and skeletal muscle (195). Supplementation with Tau prevents the development of hypertension in several animal models (196, 197). Tau has also been shown to reduce blood pressure and improve vascular function in hypertensive individuals (198). The Tau homolog, homotaurine, also exhibits beneficial effects, specifically on Parkinson's disease (199).

### Tau and Atherosclerosis

The effect of Tau supplementation in hypercholesterolemic rats (induced by a high-cholesterol diet) has previously been investigated. In this study it was found that the hypocholesterolemic effects of Tau are mediated by enhanced cholesterol degradation and the excretion of bile acid (200). In New Zealand, white rabbits, Tau was found to reduce the formation of myocardial apoptotic nuclei (53). However, the exact mechanism(s) by which this is mediated is unclear (53). Furthermore, Tau exhibits antioxidative properties in rabbits fed a high cholesterol diet (55). Tau has also been shown to reduce atherosclerotic risk in hyperlipidemic mice (57) and rabbits (201). In the latter study, Tau reduced atherosclerotic lesions in hyperlipidemic rabbits; the aortic lesions decreased by 31% and the levels of cholesteryl ester in the abdominal aorta, thoracic aorta, and aortic arch decreased by 54, 43, and 35%, respectively (201). Atherosclerosis inhibition in these rabbits may involve the antioxidant action of Tau (201). In Japanese quails fed a high cholesterol diet, Tau given as a supplement was shown to improve serum lipid levels and reduce the formation of atherosclerotic lesions (202). Tau supplementation also reduces aortic lipid accumulation in ApoE^−/−^ mice (57), and reduced the development and the progression of atherosclerosis in spontaneously hyperlipidemic mice, probably by lowering the content of oxidized substances and increasing serum HDLc, by mechanisms other than simply lowering cholesterol (203). Evidence of the anti-atherosclerotic effects of Tau in humans is lacking; however, epidemiological studies indicate that Tau intake has beneficial effects on CVD prevention. In a multicentre WHO Cardiovascular Diseases and Alimentary Comparison (WHO-CARDIAC) study carried out across 24 medical centers in 16 countries, Tau excretion in urine was measured over 5 years (52). The Japanese population exhibited the highest values recorded for 24 h urinary Tau excretion, and an inverse association between Tau levels and ischemic heart disease mortality, possibly due to their high dietary consumption of seafood, much of which contains high levels of Tau (52). In a follow-up study using 3,960 individuals from the WHO-CARDIAC Study (which included the Japanese population that consumed high levels of Tau), the Tau/creatinine (Cr), and magnesium (Mg)/Cr ratios were measured. It was found that the patients with ratios above the mean 24 h urine Tau/Cr and Mg/Cr ratios of 639.4 and 82.8, respectively, exhibited an inverse relationship with body mass index, total cholesterol in plasma, blood pressure (diastolic and systolic) and atherogenic indices (total cholesterol/HDLc), compared with the patients with lower levels of urinary Tau excretion (204). In support of these findings, a more recent 24 h nutrient analysis study by Jun and Choi similarly reported a negative association between Tau intake and atherogenic index in men, but not in women who appeared to have a positive association between Tau intake and diastolic blood pressure (205). In a human study that included smokers, it was found that Tau was positively associated with endothelial functioning (206). Recently, it was shown that Tau exerts anti-atherogenic as well as anti-inflammatory effects (lowering of C-reactive protein (CRP) and platelets) in patients with HF and a left ventricular ejection fraction of <50% (207). Tau and Mg supplementation significantly increased the endothelial progenitor cell (EPC) colony numbers (EPCs repair endothelial damage to prevent CVDs) and significantly decreased free radical levels and thiobarbituric acid scores in healthy men. The same trend was obtained in spontaneously hypertensive rats (208).

Interestingly, Tau is abundant in ECs and aids in their protection against oxidative stress, inflammation, and cell apoptosis suppression (209, 210). Tau also exerts a beneficial effect on lipid metabolism, which may have an essential role in CVD prevention (211). A study conducted on 17 patients with congestive HF, examined the effects of oral administration of Tau and coenzyme Q, reported improved systolic left ventricular functioning (54). Tau improves cardiovascular function by improving antioxidant effects, antagonism of Ang II action, and lipid profile (56). NO has a prominent role in Tau vasodilatory action in response to vasoactive agents and, consequently, blocks the effects of Ang II (56). Tau induces concentration-dependent inhibition of lysophosphatidic acid (found in human atherosclerotic plaques) and stimulates an increase in intracellular calcium (Ca^2+^) in cultured VSMCs (212). Tau may also slow the progression of atherosclerosis and reduce the oxidation of LDL (211).

Tau has essential roles in a range of processes such as cytoprotection, cell death, and survival, maintenance of Ca^2+^ homeostasis, and cell cycle regulation. Tau supplementation relieves symptoms and abnormalities of the circulatory system such as hypertension, ischemia-reperfusion injury, atherosclerosis, HF, and myocardial arrhythmias. Tau also prevents a high-fat diet from elevating the LDL and VLDL cholesterol levels. Tau is present in ECs and helps them adapt to hypotonic stress, protecting them from apoptosis (213). Worldwide epidemiological studies have also revealed the beneficial effects of Tau intake on CVD prevention. Based on human and animal data, Tau is a promising nutritional supplement. Although clinical evaluation of Tau supplementation has been limited to only a few clinical conditions, it has already been approved for use in congestive HF (54).

### Cys, Hcy, and Met

Elevated levels of Hcy are implicated in occlusive arterial disease in the heart, kidney, and brain, in addition to venous thrombosis, chronic renal failure (214), and oxidative stress (215). Moreover, hyperhomocysteinemia (identified as a risk factor for atherosclerosis) led Jacob *et al*. to investigate whether both cysteine and Hcy are associated with hyperlipidemia (58). They reported that both Cys and Hcy levels are lower in asymptomatic patients than in CVD patients. They concluded that high Cys levels are likely to be a risk factor for atherosclerosis in patients with hyperlipidemia based on plasma Cys levels being significantly lower in healthy individuals than in patients with carotid atherosclerosis. Other CVD risk factors, including smoking, hypertension, hypercholesterolaemia, hyperhomocysteinaemia, diabetes, obesity, and their combinations, are also associated with increased Cys levels (216).

Homocysteinemia is associated with arteriosclerotic plaques in cases where the enzymes involved in the converting Met to Hcy harbored mutations (217). Since Met is the only known source of Hcy, other studies examined Met intake in relation to vascular disease. Specifically, Toborek *et al*. fed 20 male New Zealand rabbits with standard chow or chow enriched with 0.3% Met for 6 or 9 months (218). They reported significant increases in levels of aortic thiobarbituric acid reactive substances and antioxidant activities, as well as typical atherosclerotic changes, such as calcification and deposition of cholesterol and intimal thickening, in the aortas of the Met-fed rabbits.

Subsequently, Julve *et al*. studied the impact of Met-induced hyperhomocysteinemia on two primary antiatherogenic functions of HDL, namely preventing LDL oxidation and inducing macrophage-specific reverse cholesterol transport (219). They fed C57BL/6NCr mice with standard chow and added 1% Met as a supplement to the drinking water of a subset of mice to induce hyperhomocysteinemia. The mice with Met-induced hyperhomocysteinemia exhibited decreased HDL-cholesterol levels and apolipoprotein A-I (by about 20%) and reduced activities for HDL-associated antioxidant enzymes, platelet activation factor acetylhydrolase and paraoxonase-1. Also, the hyperhomocysteinemic mice displayed decreased HDL-related cholesterol efflux from macrophages. Thus, the mice with Met-induced hyperhomocysteinemia were more prone to oxidation and displayed a lower capacity to protect their LDL against oxidative modification, which suggests the mechanism by which dietary Met induces hyperhomocysteinemia may facilitate the progression of atherosclerosis (219). Yang *et al*. demonstrated that ApoE^−/−^ mice fed high-Met diets develop lipid deposition in the arterial wall, and that fatty acid-binding protein (FABP4) promotes lipid accumulation in arterial endothelium. They also demonstrated that the demethylation of FABP4 is involved in Hcy-mediated atherosclerosis and that DNA (cytosine-5)-methyltransferase 1 enzyme (DNMT1) is a *FABP4* methylation regulatory factor (220). These data suggest that DNMT1 should be considered a possible therapeutic target for the treatment of Hcy-related atherosclerosis.

As Met load usually leads to high Hcy and cardiovascular effects, Selhub and Troen designed a study to determine if increased Met intake is atherogenic (221). They fed ApoE^−/−^ mice diets that satisfied three conditions:

- High Met intake with normal blood Hcy.
- High Met intake with hyperhomocysteinemia and vitamin B deficiency.
- Normal Met intake with both hyperhomocysteinemia and vitamin B deficiency.

**Figure 5:**
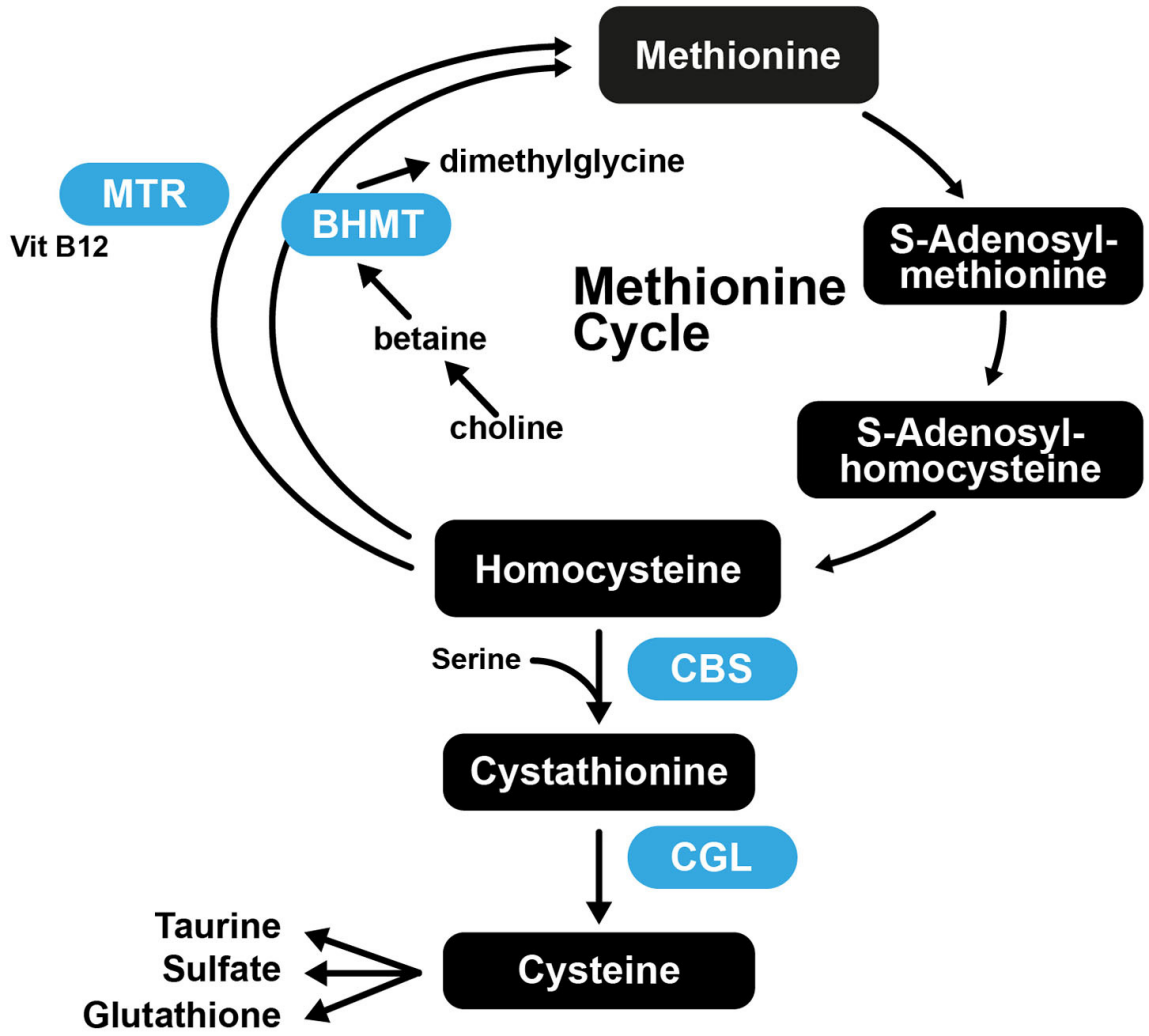
The transsulfuration pathway connecting Met and Cys biosynthesis. CBS, Cystathionine-β-synthase; CGL, cystathionine gamma-lyase; BHMT, betaine-homocysteine S-methyltransferase; MTR, 5-methyltetrahydrofolate-homocysteine methyltransferase.

It was found that mice that had normal plasma Hcy at the start of the study, that were fed Met-rich diets exhibited atheromatous effects, while mice with normal Met levels were deficient in vitamin B and developed severe hyperhomocysteinemia (221). These results suggest that the role of Hcy in arteriosclerosis is complex, as increased Met intake is atherogenic in susceptible mice, while high plasma Hcy is not. This further suggests that Cys may also not be atherogenic, as the increased Cys levels associated with atherosclerosis may merely be a consequence of Met, Hcy, and Cys being part of the transsulfuration pathway (Figure 5).

### Gly and Dimethylglycine Metabolism and Its Role in Atherosclerosis

Gly is the simplest proteinogenic AA that can be produced endogenously, but some studies indicate that its synthesis alone is insufficient to meet the metabolic needs of an organism (222). This AA is usually a constituent part of all three types of collagen (223), and elastin (224). Gly has a role in gene expression (225), glutathione synthesis (226), and low levels are associated with metabolic disorders (227, 228). Oral supplementation with Gly alleviates symptoms of several metabolic disorders (229). In humans, Gly synthesis occurs from hydroxyproline, serine, glyoxylate, and threonine primarily in the liver and kidneys (222). Approximately 2.5 g of Gly is synthesized from serine per day, which is close to the mean dietary intake of Gly (222). Gly can be catabolized back to serine (230) or can be degraded by glycine synthase via a mechanism that involves decarboxylation and deamination (231), or via a third mechanism, into glyoxalate (232). Gly exerts anti-inflammatory and antioxidative effects (233) and has been inversely associated with traditional cardiovascular risk factors, such as obesity (234), hypertension (235), and diabetes mellitus (236).

The anti-inflammatory activities of Gly are most probably the result of a decrease in macrophage and leucocyte signaling and the synthesis of inflammatory mediators. A decrease in cell signaling may originate from glycine-gated chloride channel-induced hyperpolarization at the cell membrane. This could explain its protective effects on coronary arteries, accompanied by an increase in eNOS and NO bioavailability (237). CHD risk inversely correlates with serum levels of Gly, independently of sex (59, 238). Concerning its cardiovascular activities, the cardioprotective effects of Gly are mediated via activation of Gly receptor α2 (GlyRα2) in cardiomyocytes, which promotes attenuation of cardiac hypertrophy and fibrosis in animal models (238). Low Gly levels are linked to IR and type 2 diabetes. Gly stimulates insulin secretion by binding to GlyRα1 on pancreatic β cells (238, 239). The antiatherogenic potential of Gly has been examined by monitoring the levels of Gly and the expression of *APOA1BP*, the gene that encodes apoA1-binding protein. APOA1BP is involved in HDL-mediated (reverse) cholesterol transport from peripheral tissues to the liver (240). Gly levels positively correlate with serum HDL-C and apoA1 levels and are inversely correlated with triglyceride, CRP and apoB levels, and cIMT (238, 240). Additionally, Gly inhibits cellular VLDL uptake and decreases triglyceride synthesis (238). Gly exerts its antioxidative effects by reducing through oxidative stress components and restoration of glutathione biosynthesis, a major antioxidant in human vascular tissues. The underlying mechanisms that mediate such effects involve reducing NADPH-oxidase activity and increasing copper (Cu), Zn-SOD activity (241).

Rom *et al*. studied the effects of supplementation with 0.75% Gly in the drinking water of ApoE^−/−^ mice for 40 days. They reported that triglyceride and aortic cholesterol mass, and aortic lipid peroxides, have a decreasing trend following supplementation (242). Gly exerts protective effects, including immunomodulatory, anti-inflammatory, and direct cytoprotective actions. Gly suppresses free radical formation, transcription factor activation, and inflammatory cytokines by acting on inflammatory cells such as macrophages (243). In a rat model, it was found that intraperitoneal administration of Gly (0.5 mg/g body weight) reduces cardiac ischemia/reperfusion injury and myocardial apoptosis, with the ischemic area significantly decreased (244). This study suggests that Gly is a reliable therapeutic agent that can ameliorate heart failure after MI (244). In rat cardiomyocyte cultures (HL-1), the addition of Gly (3 mM) during re-energization completely prevented necrotic cell death associated with pH normalization an hour after simulated ischemia (245). Gly supplementation has a positive effect on cardiomyocyte survival after an ischaemic insult and could represent a promising therapeutic approach to prevent cell death in reperfused myocardium (245).

In mice, it was shown that Gly (at a dose of 700 mg/kg) attenuates left ventricular hypertrophy and cardiac fibrosis induced by either transverse aortic constriction or Ang II administration, potentially via inhibition of extracellular signal-regulated kinase phosphorylation and the reduced synthesis of transforming growth factor-β and ET-1 in cardiomyocytes (246). Human platelets are Gly-responsive and express Gly-gated chloride channels. Gly prevents aggregation of platelets, a process central to clot formation (62). Gly supplementation (1% Gly added to the drinking water) was examined in rats after sucrose ingestion. It was found that Gly decreased IR and the levels of various oxidative stress markers (247). Associations between plasma Gly and the incidence of acute MI (AMI) in a cohort of patients with suspected stable angina pectoris show Gly is inversely associated with several CAD risk factors (248).

Gly exhibits notable roles in a wide range of processes, including cell signaling pathways. Current evidence suggests that Gly supplementation may offer a therapeutic benefit to some patients, particularly following ischaemic events. Dimethylglycine (DMG) is a derivative of Gly with the structural formula (CH3)_2_NCH_2_COOH. Dietary sources of DMG include beans and liver, but DMG can also be synthesized from betaine during Hcy remethylation to Met (249). This reaction is catalyzed by the enzyme, betaine-homocysteine methyltransferase, which is found predominantly in the liver and kidneys (250). DMG is metabolized to sarcosine, providing a substrate for the synthesis of 5,10-methylenetetrahydrofolate (251). A smaller proportion of DMG is excreted unmetabolized in the urine (252). A study involving LDL receptor-deficient mice fed a high-fat diet showed that DMG perturbed the tricarboxylic acid cycle, fatty acid metabolism, choline metabolism, and markedly reduced excretion of urine due to the lower recycling rate of choline-containing metabolites (253). Plasma DMG concentrations were observed to be elevated in patients with chronic renal failure, based on a study involving 33 dialysis patients, where DMG levels positively correlated with Hcy levels, which present independently of other atherosclerotic risk factors (254).

DMG levels are strongly and independently associated with the risk of future AMI in patients with stable angina pectoris (61). Furthermore, there is a causal relationship between DMG and stable angina pectoris and AMI. It has been shown that plasma DMG improved risk prediction for all-cause cardiovascular mortality in a group of patients with AMI (255). In both children with congenital heart defects and their mothers, serum or plasma concentrations of DMG are higher compared with controls. Elevated DMG in the disease group may indicate the upregulation of the betaine homocysteine methyltransferase pathway (256). Low levels of plasma DMG are associated with a lower bone mineral density and an increased risk of hip fracture (257). Based on these studies, increased plasma DMG levels are associated with atherosclerotic CVD, whereas elevated Gly has positive effects on atherosclerosis and associated disorders.

## Immunomodulation of Atherosclerosis

Atherosclerotic plaques contain a variety of ICs, including T cells, macrophages, dendritic cells, neutrophils, and natural killer cells (258). As mentioned above, monocytes infiltrate the intima and transform into macrophages that engulf oxLDL and apoptotic cells, which leads to their transformation into foam cells (64). These macrophages polarize into L-Arg, h-Arg, and Trp metabolizing subtypes. *In vivo* studies were used to demonstrate the induction of ARG1 and SLC7A1 (CAT) transporter expression in myeloid-derived suppressor cells. Also, based on gene expression data, neutrophils express a relatively low number of SLC7A (CAT) transporters, which import extracellular Arg but highly express ARG2, which will facilitate Arg catabolism. This observation suggests that neutrophils can deprive their environment of Arg, which may affect surrounding cells (259). Macrophages are similarly able to import AAs to influence the function of neighboring cells. Macrophages express all major enzymes involved in L-Arg, h-Arg, and Trp metabolism, including ARG1, iNOS, and IDO-1, which collectively arm macrophages with various immune regulatory capacities. Specifically, in a mouse model (Female C57BL/6), M1 macrophages are classified based on their expression of iNOS, whereas M2 macrophages characteristically express ARG1 (260). Macrophage differentiation is cytokine-induced, suggesting that plaque-laden macrophages form clusters of particular subsets dependent on their local environment. For example, it is known that the expression of iNOS and ARG1 in macrophages is induced by Th2-cytokines (64).

### Macrophages

In 1979, Gerrity *et al*. were the first to report that macrophages constitute a large portion of porcine lesions (261). Today, we know that macrophages release inflammatory cytokines, play a role in vascular remodeling (262), and are transformed into foam cells. The uptake of lipids by macrophages is enhanced after stimulation of Toll-like receptors (TLRs) TLR2, TLR4, and TLR9 by corresponding ligands, making TLRs important for atheroma development. Intracellular lipid accumulation by foam cells in the arterial walls is enhanced after stimulation by pro-inflammatory molecules (263). Thus, atheroma development is dependent on the activity of TLRs (263, 264). Macrophages possess TLR3, TLR4, and TLR9 receptors, which stimulate the expression of Scavenger receptors that, in turn, facilitate LDL intake (265), and suppress cholesterol efflux from macrophages (266).

As previously mentioned, macrophage phenotypes can be altered in response to various signals (267). The two major macrophage subtypes are M1 (pro-inflammatory) and M2 (tissue homeostasis and repair). These subtypes play opposing roles but are both found in atherosclerotic plaques. The classical M1 phenotype is induced by inflammatory cytokine signaling [such as interferon-γ (IFN-γ)], tumor necrosis factor-α (TNF-α) and invading pathogens (268, 269). M1 macrophages release inflammatory cytokines, IL-1β, IL-23, IL-12, IL-6, and TNF-α and chemokines, CXCL11, CXCL10, and CXCL9 (270). M1 cells participate in Th1-mediated immune responses and produce high levels of NO and ROS. By contrast, M2 macrophages are involved in Th2-dependent immune cascades and play an anti-inflammatory role. Th2-type cytokines induce the formation of M2 macrophages, which release the anti-inflammatory cytokine, IL-10 (270). Unique macrophage subtypes play anti-inflammatory, antioxidant and atheroprotective roles in hemorrhagic atherosclerotic plaques (258).

Extensive cellular metabolic changes accompany macrophage differentiation. Pro-inflammatory M1 macrophages utilize L-Arg to produce NO, which is catalyzed by iNOS. On the other hand, activated M2 macrophages use a different metabolic route for L-Arg, that is, L-Arg is processed to ornithine and then in turn into L-proline via the enzymes ARG and ornithine aminotransferase (271).

M2 macrophages metabolize glucose through oxidative phosphorylation, while M1 macrophages are dependent on glycolysis (271). Macrophage differentiation also alters cholesterol metabolism during atherosclerosis development (272). The pro-inflammatory properties of the atherogenic modified LDL can influence the monocyte/macrophage phenotype by altering cell metabolism. Stimulation of myeloid cells with modified LDL brings about changes in cholesterol metabolism and glycolysis rate relative to oxidative phosphorylation (272, 273). This effect has been demonstrated both *in vitro* and *in vivo* using animal models. Thus, atherosclerosis development is highly dependent on the balance between pro- and anti-inflammatory differentiation of macrophages. Consequently, statins that normalize cholesterol metabolism and antioxidants that counteract ROS formation are being used to modulate macrophage metabolism to treat atherosclerosis (258).

Both pro- and anti-inflammatory macrophages are found in atherosclerotic plaques, and the balance between these at the arterial wall is tightly linked to atherosclerotic lesion progression (274). Each macrophage subset differs in its localization at the lesion and its relative abundance. Specific macrophage subtypes are responsible for heme cleaning at sites of intraplaque hemorrhage. The M1 macrophages that trigger plaque destabilization and promote thrombus formation are localized in the plaque lipid core in humans but are distributed across the lesion in advanced murine plaques (275, 276). By contrast, the M2 macrophages, which are present in human and murine plaques (275, 276), contain smaller lipid droplets compared with M1 macrophages and the M2 macrophages, which surround the lipid core (275). M2 macrophages function in the recruitment of fibroblasts, wound healing, and tissue repair through matrix remodeling and also potentially in apoptotic cell clearance within the plaque (274).

### Role of IDO

IDO is an important element of various pathological conditions due to its immunoregulatory features. IDO-1 expression and activity in numerous cell types, such as cancer, immune, endothelial, and smooth muscle cells are controlled by inflammatory processes (277). The main mechanisms by which IDO modulates immunity are via Trp depletion, stimulation of the stress sensor General Control Nonderepressible 2 (278), and regulation of the KYN pathway via bioactive metabolite production (279).

Since inflammation is an integral part of atherosclerosis, various experimental models have demonstrated that modulation of innate or adaptive immune responses is effective against atherosclerosis (280–282). Inflammation induced by atherosclerosis is controlled through the release of Th1-type cytokines, specifically, IFN-γ (283), which induces IDO expression. Further to this, IDO deficiency dysregulates cytokine IL-10 release and promotes early-stage atherosclerosis (110). These data suggest that IDO, through its immune-inflammatory actions, may be a promising therapeutic agent against atherosclerosis (284).

IDO inhibition with 1-methyl Trp in ApoE^−/−^ mice led to aberrant lipid handling and enhanced vascular inflammation (285). Moreover, elevated IDO-1 is correlated with a reduction of human and mouse atherosclerosis, where plasmacytoid dendritic cells (pDCs) overexpress IDO-1 and modulate T cell responses (286, 287). IDO immunomodulatory responses in atherogenic plaques, precisely IDO-1 expression, are also associated with ECs and VSMCs (288–290). Furthermore, induced IDO-1 activity in cultured human VSMCs treated with IFNγ, suppressed T cell accumulation, activation, and proliferation (290). In hypercholesterolemic mice, the genetic ablation of IDO-1 enhances vascular IL-10, confers atheroprotection and IDO-dependent immunoregulatory responses (112). Therefore, IDO-1 stimulated expression has various actions on the immune system and might be effective against inflammation (279). For instance, eicosapentaenoic acid administration in LDLr^−/−^ mice stimulates IDO-1 expression and reduces vascular inflammation and atherosclerosis, possibly through decreasing the numbers of macrophages, DCs, and T cells (291–293). Regulatory T cells participate in atherosclerosis by promoting plaque stabilization (294) and influence inflammation by inducing IDO-1 expression in antigen-presenting cells (295). IDO-1 stimulation may however, lead to undesired effects, such as defective immunity and increased susceptibility to infection (279). For example, IDO has been shown to promote infection by reducing host protective immunity (296).

### Role of ARGs

ARG is an enzyme that regulates the bioavailability of NO by inhibiting competitively with eNOS. ARG1 and ARG2 are two isoforms of ARG. Two separate genes encode the arginase isoenzymes, and their products have different lengths (ARG1 has 322 AAs, whereas ARG2 has 354 AAs) (297, 298). The two isoenzymes occupy different intracellular locations as well: ARG1 resides in the cytoplasmic compartment, whereas ARG2 is localized in the mitochondria (299). Human ARG1 shares a sequence identity of 58% to human ARG2 (300). These two isoenzymes catalyze the same reaction, but their function depends on where they are localized within a particular tissue or cell. Elevated activity of ARG isoforms in the endothelium diminishes the vasoprotective role of NO (301), whereas these two isoforms have opposite roles in macrophages (302).

#### ARG1

ARG1 is an essential immunological regulator in macrophages. It catalyzes L-Arg hydrolysis to form urea and L-ornithine (a polyamine precursor) and L-proline. Thus, ARG1 activity represses the NO-mediated cytotoxicity via L-Arg consumption (300). Substrate competition between iNOS and ARG1 occurs in mouse macrophages (300), but further studies are required to confirm that this also happens in human macrophages. The function of NO in human macrophages is controversial because human monocyte-macrophage cells cannot be easily stimulated to produce iNOS or NO (303). However, there is evidence that human tissues express iNOS (304). In humans, ARG1 is most highly localized within the granulocytes of a subpopulation of myeloid cells (polymorphonuclear leukocytes) (305).

Macrophage ARG1 may slow down the progression of atherosclerotic plaques (306). Both human (307) and mouse (276, 308–310)—focused studies have reported ARG1 expression in atherosclerotic plaques. Wang *et al*. (311) used New Zealand rabbits to demonstrate that elevated ARG1 levels competitively inhibit iNOS to stimulate an anti-inflammatory response, which increases the release of Th2-type cytokines. They also found that ARG1 provides a further protective role in atherosclerosis by increasing intracellular polyamine production, and in VSMCs, the generation of collagen, which thickens the fibrous cap to provide stable plaques (276, 311). In two strains of rabbits (with low or high atherosclerosis resistance), elevated levels of ARG1 in macrophages were demonstrated to lead to atherosclerosis (312).

#### ARG2

Ryoo *et al*. studied the role of ARG2 in the development of atherosclerosis and reported that OxLDL stimulates ARG2 release and reduces NO production (313). They also reported that the inhibition of endothelial ARG or the deletion of the ARG2 gene restores endothelial function, and reduces plaque burden. These data imply that ARG2 should be a target for the treatment of atherosclerotic vascular disease. Thus, the endothelium is protected through the genetic inhibition of ARG (314). The idea that genetic inhibition of ARG may be beneficial was confirmed by the repression of ARG2 gene expression in a murine study (315). In animal models with atherosclerosis, ARG2-dependent increases in ROS can lead to endothelial cell dysfunction (316). Xiong *et al*. have recently used human umbilical veins to demonstrate that activated ARG2 potentiates proliferative processes in vascular cells (317). Also, Ming *et al*. used atherosclerosis mouse models with a deficiency in ARG2 to demonstrate the protective effects (of reduced ARG2) against atherosclerosis from an inflammatory perspective (318). Afterward, increased activity of ARG2, irrespective of plasma lipid levels, was sufficient to induce inflammatory changes and atherosclerosis formation (319).

ET-1 exerts proinflammatory effects through endothelin receptors A and B, present in the VSMCs. In an atherosclerotic mouse model, blocking ET-A receptors attenuated atherosclerosis (320), and ET-1 has been associated with human atherosclerosis experimental models (321). The interplay between ET-1 and ARG expression and activity has been explored in a study by Rafnsson *et al*. (322). They report results on human carotid artery ECs, and THP-1 monocyte cells (a spontaneously immortalized monocyte-like cell line), which show that ET-1 and its receptors are expressed in the macrophages and ECs of human carotid plaques. Specifically, ET-1 co-localizes with ARG1 and ARG2. The same report also demonstrated that ET-1 stimulates ARG expression, as well as ARG activity in both THP-1, derived macrophages and ECs. ET-1 also stimulates the formation of ROS through a mechanism dependent on ARG. All this evidence indicates that in later stages of atherosclerosis, there is a significant relationship between ET-1 and ARG (322).

## Conclusion

In this review, we have summarized our current knowledge of essential, conditionally essential, and non-essential AAs in atherosclerosis and atherosclerosis-related CVDs. At present, the weight of evidence indicates that catabolic defects in BCAA metabolism can lead to elevated plasma BCAA concentrations, representing risk factors for cardiometabolic diseases. Alterations in the levels of BCAAs are associated with disorders, including renal failure, atherosclerosis, and cancer (79). Reduced plasma Trp levels are related to various disease states. Patients with atherosclerotic plaques have lower serum Trp levels compared with healthy controls. Further work is required to understand whether Trp supplementation may represent a therapeutic (or preventative) strategy to improve cardiovascular health. Patients with atherosclerosis and related complications in some studies show benefits from L-Arg administration and in others not. The optimum oral dosage of L-Arg also remains to be established in these and other diseases associated with NO-related dysfunction. The controversial effects of L-Arg are probably due to variations between patients and their metabolism, which can generate compounds that are inhibitors of NOS. More studies are required before identifying those individuals who would benefit from L-Arg and those who would not.

The weight of evidence suggests that h-Arg may be more than merely a marker of adverse outcomes, but that it plays a direct protective role in cardiovascular and metabolic pathologies (175). Worldwide epidemiological studies have also revealed the beneficial effects of Tau intake on CVD prevention. Based on human and animal data, Tau is a promising nutritional supplement. Although Tau's clinical evaluation has been limited to a few disorders, it has already been approved for congestive heart failure. Gly is a simple AA that is important for cardiovascular health. It plays notable roles in a wide range of processes, including cell signaling pathways. Gly plays prominent roles in a wide range of pathologies, among which are following ischemic events, and its role in lowering the lipid status is known. Current evidence suggests that Gly supplementation may offer a therapeutic benefit to some patients, particularly following ischemic events. In contrast, elevated levels of the Gly-derivative, DMG are associated with atherosclerosis and related disorders. Elevated Cys in circulation correlates with CVD risk factors. Collectively, AAs appear to represent important CVD risk indicators, and further work is required to fully understand their involvement in such pathologies.

Today, the immunomodulation of atherosclerosis is at the center of current research. Available data suggest that IDO regulation is mediated through various immunological signals (288), and also suggest that IDO is a promising therapeutic agent against atherosclerosis (284). Similarly, ARG inhibitors have been considered and evaluated as promising therapeutic agents against atherosclerosis and other CVDs since the 1990s (301, 323).

These studies have yielded promising results in terms of improving endothelial functioning. However, we still do not have specific inhibitors for each isoform, limiting our understanding as we do not know which isoforms are responsible for particular observed effects. Innovative therapeutic strategies for atherosclerosis prevention and treatment through immunological induction of atheroprotective mechanisms have been proposed and are desirable (6, 324, 325). Data supporting a role for immune dysregulation underlying atherosclerosis are now coming to light and are expected to yield novel therapeutic targets.

## Author Contributions

BZ and EI contributed to the conceptualization of this work, wrote the article, and reviewed the manuscript. JR reviewed the literature and reviewed the article. ME, OM, and TG contributed to the conceptualization of the article and wrote the first draft of sections of the article. AS and ZG critically read the manuscript and corrected the language. All authors reviewed the final draft.

## Conflict of Interest

The authors declare that the research was conducted in the absence of any commercial or financial relationships that could be construed as a potential conflict of interest.

## Abbreviations

AAs: Amino acids
ADMA: Asymmetric dimethylarginine
AG: Agmatine
AGAT: Arginine glycine amidinotransferase
AMI: Acute myocardial infarction
Ang: Angiotensin
apoB: Apolipoprotein B
ApoE^−/−^: Apolipoprotein E null mice
ARG: Arginase
Arg: Arginine
BAT: Brown adipose tissue
BCAAs: Branched-chain amino acids
BCAT: Branched-chain amino acid aminotransferase
BCKD: Branched-chain α-ketoacid dehydrogenase
CAD: Coronary artery disease
CHD: Coronary heart disease
cIMT: Carotid intima-media thickness
CoA: Coenzyme A
Cr: Creatinine
CRP: C-reactive protein
CVDs: Cardiovascular diseases
Cys: Cysteine
DCs: Dendritic cells
DDAH: Dimethylarginine dimethylaminohydrolase
DMG: Dimethylglycine
DNMT1: DNA (cytosine-5)-methyltransferase 1 enzyme
ECs: Endothelial cells
eNOS: Endothelial nitric oxide synthase
EPCs: Endothelial progenitor cells
ET-1: Endothelin-1
FABP4: Fatty acid-binding protein gene
Gly: Glycine
GlyRα2: Gly receptor α2
HAA-: 3-hydroxyanthranilic acid
h-Arg: Homoarginine
Hcy: Homocysteine
HDLc: High-density lipoprotein cholesterol
HF: Heart failure
ICs: Immune cells
IDO: Indoleamine 2,3-dioxygenase
IFN-γ: Interferon gamma
Ile: Isoleucine
iNOS: Inducible nitric oxide synthase
IR: Insulin resistance
KA: Kynurenic acid
KP: Kynurenine synthesis pathway
KYN: Kynurenine
L-Arg: L-Arginine
LDL: Low-density lipoprotein
Leu: Leucine
Met: Methionine
Mg: Magnesium
MI: Myocardial infarction
NO: Nitric oxide
NOS: Nitric oxide synthase
nNOS: Neuronal nitric oxide synthase
OxLDL: Oxidized low-density lipoprotein
pDCs: Plasmocytoid dendritic cells
PUTR: Putrescine
ROS: Reactive oxygen species
SAM: S-adenosyl methionine
SLC25A44: Carrier Family 25 Member 44 gene
SOD: Superoxide dismutase
Tau: Taurine
TDO2: Tryptophan 2,3 dioxygenase
TLRs: Toll-like receptors
TNF-α: Tumor necrosis factor alfa
Trp: Tryptophan
Val: Valine
VLDL: Very low density lipoprotein
VSMCs: Vascular smooth cells
WHO: World Health Organization
Zn: Zinc
3-HIB: 3-Hydroxyisobutyric acid
5-HT: Serotonin.
